# Carbohydrate Immune Adjuvants in Subunit Vaccines

**DOI:** 10.3390/pharmaceutics12100965

**Published:** 2020-10-14

**Authors:** Sahra Bashiri, Prashamsa Koirala, Istvan Toth, Mariusz Skwarczynski

**Affiliations:** 1 School of Chemistry and Molecular Bioscience, The University of Queensland, St Lucia, Brisbane, Queensland 4072, Australia; s.bashiri@uq.edu.au (S.B.); p.koirala@uq.edu.au (P.K.); 2 Institute for Molecular Bioscience, The University of Queensland, St Lucia, Brisbane, Queensland 4072, Australia; 3 School of Pharmacy, The University of Queensland, St Lucia, Brisbane, Queensland 4072, Australia

**Keywords:** carbohydrates, peptide/protein subunit vaccines, immunostimulation, adjuvants

## Abstract

Modern subunit vaccines are composed of antigens and a delivery system and/or adjuvant (immune stimulator) that triggers the desired immune responses. Adjuvants mimic pathogen-associated molecular patterns (PAMPs) that are typically associated with infections. Carbohydrates displayed on the surface of pathogens are often recognized as PAMPs by receptors on antigen-presenting cells (APCs). Consequently, carbohydrates and their analogues have been used as adjuvants and delivery systems to promote antigen transport to APCs. Carbohydrates are biocompatible, usually nontoxic, biodegradable, and some are mucoadhesive. As such, carbohydrates and their derivatives have been intensively explored for the development of new adjuvants. This review assesses the immunological functions of carbohydrate ligands and their ability to enhance systemic and mucosal immune responses against co-administered antigens. The role of carbohydrate-based adjuvants/delivery systems in the development of subunit vaccines is discussed in detail.

## 1. Introduction

Subunit vaccines built from fragments of pathogens, such as proteins, are becoming extensively popular in vaccine design [1]. In contrast to classical vaccines based on whole organisms (live and attenuated pathogen), subunit vaccines avoid the use of redundant pathogen components, greatly reducing the likelihood of triggering autoimmunity or allergic responses [2,3]. Synthetic peptide/protein subunit vaccines can be designed to induce only the desired immune responses. However, peptides/proteins as antigens, alone, are poorly immunogenic as they are not easily recognized by immune cells. They have a low permeability and oral absorption due to their high molecular weight and hydrophilic character [4]. In addition, both proteins and peptides are susceptible to enzymatic degradation, conferring short half-lives *in vivo.* Consequently, subunit vaccines need the assistance of adjuvants and/or delivery systems [2].

Carbohydrates are natural and anabolic products present in all living organisms, including animals, plants, fungi, and bacteria. They exist in a variety of forms, such as monosaccharides, oligosaccharides, polysaccharides, and glycoconjugates (e.g., glycoproteins, glycolipids). While they are responsible for critical biological functions in the healthy human body, they also have roles in pathogenesis, such as microbial adherence, colonization, biofilm formation, and virulence [5].

Carbohydrates on bacterial cell surfaces are capable of adhering to human tissue, preventing bacteria from desiccation, protecting them against complement deposition, and providing protection against innate defense mechanisms. Certain structures of carbohydrates are also associated with pathogen invasion. However, pathogen-associated carbohydrates are often recognized by the host’s immune system and signal the pathogen as a foreign invader [6]. As most vaccine strategies are designed to mimic the key features of pathogens, carbohydrates can be used as natural and relatively safe vaccine adjuvants/immune stimulators, recognized by receptors on the surface of antigen-presenting cells (APCs). Following recognition, they can initiate a cascade of innate and adaptive immune responses [7,8,9,10,11]. Moreover, carbohydrate (e.g., chitosan, glucan, hyaluronic acid)-based delivery vehicles can transport antigens to desired sites in the body, such as local lymph nodes. They are especially efficient for mucosal antigen delivery because of their mucoadhesive properties [9,12]. Due to the generally inherent immunomodulating properties, biocompatibility, biodegradability, and low toxicity of carbohydrates, they are becoming intensively studied as adjuvants and delivery systems [13].

Since the antigenic function of carbohydrates is already extensively covered elsewhere [6,8,14], this review focuses on the receptor-based recognition of carbohydrates and their ability to enhance systemic and mucosal immunity against co-administered antigens. Similarly, the gene-based (DNA/mRNA) subunit vaccines have been recently reviewed [15,16], and therefore this review emphasizes the protein/peptide-based subunit vaccines.

## 2. Immunostimulation

### 2.1. Innate and Adaptive Immunity

To design an effective vaccine, an understanding of how the vaccine will interact with the immune systems, as well as the nature of the immune systems itself, is crucial. Innate and adaptive immunity are the two main components of immune systems. Innate immunity refers to the nonspecific defense mechanisms of the body that are activated immediately after pathogen attack. Innate immunity keeps foreign particles out of the body or limits the ability of the foreign material to spread and move throughout the body. This occurs through a variety of mechanisms, including physiological and anatomical barriers (e.g., skin), inflammatory responses, secretion of antimicrobial peptides, and phagocytosis [17]. The innate immune system is also responsible for activating the adaptive immunity. Adaptive immunity provides antigen (pathogen)-specific responses, including long-lasting memory immune responses. Adaptive immune responses are divided into cell-mediated and humoral immunity.

**Figure 1:**
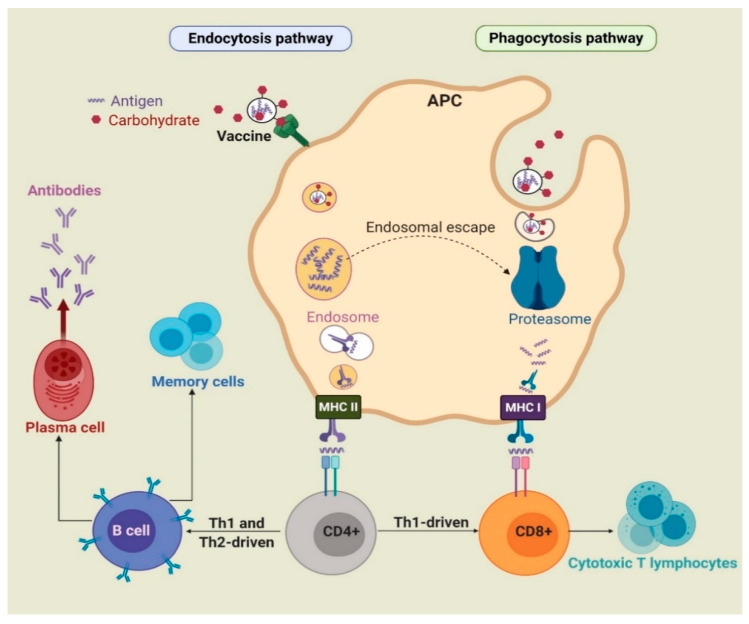
The basic uptake pathways of antigens (e.g., endocytosis and phagocytosis) by antigen-presenting cells (APCs) to induce immune responses.

During infection or following vaccine administration, antigens are first taken up by immature APCs, such as macrophages and dendritic cells (DCs), with the help of pattern recognition receptors (PRRs), such as toll-like receptors (TLRs) and C-type lectin receptors (CLRs). APCs then mature and the captured antigens are degraded to peptide fragments in endosomes or by proteasome and recognized by major histocompatibility complex (MHC) class II or class I molecules, respectively. The MHCs/peptide complexes are then transported on the surface of APCs, where they can be recognized by T-cell antigen receptors (TCRs). CD8^+^ and CD4^+^ T-cells are stimulated by antigen-MHC I and II complexes, respectively. Upon activation, CD8^+^ cells change into cytotoxic T-lymphocytes (CTLs), which directly kill infected cells. Activated CD4^+^ cells differentiate into either T-helper 1 (Th1) or T-helper 2 (Th2) cells [2,18]. Th1 cells produce cytokines, such as interferon-γ (IFN-γ), tumor necrosis factor-α (TNF-α), and interleukin (IL-2 and IL-12) and stimulate mainly cell-mediated immunity (CD8^+^ cells) against intercellular pathogens [3]. Cytokines produced by Th2 include IL-1, IL-4, IL-5, IL-6, IL-10, and IL-13. In addition, upon antigen (B-cell epitopes) recognition, B-cells are stimulated by Th2 cells to differentiate into memory and plasma cells and produce antibody-based humoral immune responses (Figure 1) [19]. As such, B-cells are part of the adaptive immune response and contribute to immunological memory, the process whereby immune cells respond more quickly and efficiently to an antigen that they have encountered previously [20].

Carbohydrates can be recognized by receptors present on the surface of APCs and, when associated with an antigen, can enhance uptake via endocytosis/phagocytosis. For example, mannose can be recognized by mannose receptor [21]; chitosan can bind to different receptors, including TLR2 and TLR4 [22]; β-glucan can be recognized by receptors consisting of dectin-1, TLR2, 4, and 5 [23]; lipid A, a lipidated disaccharide, can be taken up by TLR4 [24]; and saponin can bind to DC-SIGN receptors [25].

Furthermore, carbohydrate incorporation within nanovaccines can activate cross-presentation of antigens through phagocytosis, endosome escape of antigen and lysosomal processing, and finally the MHC I pathway [26]. Antigens alone are usually unable to activate the immune system due to low capacity, to be taken up by DCs and to reach the MHC I processing pathway [27]. However, sufficient uptake of antigens by APCs facilitated by carbohydrate-based adjuvants initiates processing and presentation by MHC I and triggers the physiological processes involved in cross-presentation and CTL priming [28]. These responses are essential for triggering antitumor immunity.

### 2.2. Mucosal and Systemic Immunity

Human immune responses can be divided into systemic and mucosal immunity. The sterile interior cavity of the body is known as the “systemic” environment, and it contains all of the organs except for the skin and mucosal surfaces. Injection into the body is needed to stimulate the systemic immune system and result in the production of protective antibodies and T-cells. Therefore, the first interaction between an infectious agent and a host usually occurs on a mucosal surface. Thus, mucosal immunity can prevent the entrance of pathogens into the body and stop resulting infection. The mucosal immune system consists of an integrated network of tissues, lymphoid and constitutive cells, and effector molecules (antibodies, cytokines, and chemokines) [29]. As such, mucosal infection, or a mucosal-administered vaccine, can result in the activation of protective B- and T-cells in both mucosal and systemic environments [30].

**Figure 2:**
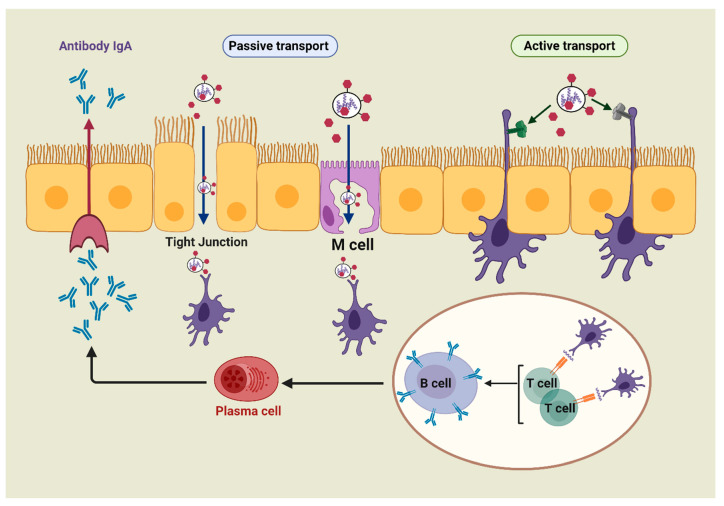
The simplified pathway of mucosal immunity.

The mucosa-associated lymphoid tissue includes the nasal-associated lymphoid tissue (NALT), the bronchus-associated lymphoid tissue (BALT), and the gut-associated lymphoid tissue (GALT) [31]. All these tissues serve as the principal mucosal inductive and effector sites. The inductive sites include mucosa-associated follicles such as intestinal Peyer’s patches (PPs), isolated lymphoid follicles, and lymphoid aggregation in the bronchial tract [32]. In addition, these sites comprise a complex network of innate and adaptive immune components, such as DCs, macrophages, T-cells, B-cells, and natural killer (NK) cells, which are overlayered with epithelial cells and specialized microfold cells (M-cells) [33]. M-cells offer functional openings in the epithelial barrier through vesicular transport activity, and the ability of antigens to release the intraepithelial space, facilitating antigen contact with T and B lymphocytes and macrophages [30,34]. Besides M-cells, the antigen can be collected by the receptors present on DC transepithelial dendrites (active transport). By taking up the antigen, the antigen-specific T- and B-cells undergo activation and differentiation into T and B effector cells, which results in secretion of immunoglobulin A (IgA). The effector cells and their actions (e.g., antibody production, cytokine secretions, and cytotoxicity) migrate from inductive sites to effector sites (e.g., lamina propria and surface epithelium). Therefore, the immune responses of mucosal systems operate on the same general principle of systemic immunity: capturing the antigen with DCs or macrophages, then inducing T-cell responses and/or generating antibodies (Figure 2).

Targeting the mucosal routes (e.g., oral, nasal, pulmonary, ocular, rectal, vaginal, etc.) has several advantages over parenteral vaccination, including: (a) injection can be avoided, reducing the risk of needle-stick injury and cross-contamination [35]; (b) full sterilization of the vaccine is not required; (c) the vaccine can be self-delivered; (d) vaccine storage conditions are usually less restrictive [36]; (e) immunization of one mucosal site often induces immune responses in other mucosal effector tissues [37]; and (f) most importantly, the production of mucosal antibodies (IgA) can prevent systemic infection [30,38].

Nanoparticles based on polysaccharides can interact with M-cells and undergo endocytosis and transcytosis [39,40]. Carbohydrates, for example, chitosan-based carriers, can open tight junctions to directly transfer antigens through epithelial barriers [30]. These carriers also provide a prolonged interaction between associated antigens and epithelial cells due to chitosan’s mucoadhesive properties. Consequently, they elicit strong systemic immunoglobulin (IgG) and mucosal IgA responses [41].

## 3. Adjuvants

Adjuvants are defined as any substances, compounds, or even strategies that result in the enhancement of immune responses, particularly adaptive immune responses, when delivered together with an antigen [42,43]. An adjuvant is primarily used to mimic the danger signal and is recognized by PRRs to facilitate antigen uptake via major target cell types (e.g., monocytes, macrophages, and DCs), thus enhancing immunogenicity. In addition, adjuvants can activate and maturate macrophages, T lymphocytes, B lymphocytes, and NK cells [13,42], and inflammasomes, pro- and anti-inflammatory signals, inducing inflammatory chemokine and cytokine responses [5,44,45,46].

The key features of a successful adjuvant include not only its immune-stimulating potency to reduce the dose of antigens and the number of boost immunizations needed to trigger long-lasting protective immunity, but also its safety and stability. Salt-based adjuvants such as aluminum have been introduced as an adjuvant for human use since the 1930s [47]. Although it stimulates effective humoral immune responses against whole pathogen-based vaccines, its stimulating potency against subunit vaccines is limited. Alum is a poor immune stimulator of CTLs and induces side reactions such as indurations, erythemas, cutaneous nodules, swellings, irritation, and redness [48]. More recently, several other adjuvants have been developed; however, they have been approved only for the use in particular vaccines and countries [49]. Therefore, the development of a safe and effective adjuvant is still of high priority.

Carbohydrates are promptly recognized APCs, can protect antigen against degradation, have mucoadhesive properties and most importantly are usually biodegradable and safe for human use. Therefore, carbohydrates, both natural and synthetic, are one of the most promising and most investigated immune stimulators.

## 4. Carbohydrate-Based Adjuvants

Carbohydrates enhance the immunogenicity of a vaccine by binding to specific glycan-binding receptors on the surface of APCs (e.g., TLRs, nucleotide-binding oligomerization domain 2 (NOD2)-like receptors (NLRs), and CLRs). This encourages carbohydrate and associated antigen uptake by phagocytosis and endocytosis [50].

Specifically, CLRs, including mannose receptors, DEC-205, dectin-1, DNGR-1, DC-SING, Mincle, and single CD22, have the potential to recognize carbohydrate (sugar) moieties of glycosylated antigens [9,25,51,52]. In addition to CLRs, T-cell receptors, such as TLR2 and TLR4, can recognize pathogen-derived polysaccharides and glycoconjugates [43,53,54]. Carbohydrate interaction with CLRs triggers intracellular signaling cascades mediating APC maturation, which is necessary for naive B- and T-cell activation [55,56]. In addition, carbohydrates can also be taken up by APCs based on charged interactions [57]. Cumulatively, this triggers a variety of signaling pathways resulting in the stimulation of innate and adaptive immune responses [9]. Importantly, carbohydrates can stimulate balanced pro- and anti-inflammatory responses generating improved potency and safety compared to other adjuvants [55].

Carbohydrates with adjuvating activities are generally divided into two classes: saccharides, such as mannose and mannan; and saccharide derivatives, which are sugars modified with lipids, peptides, etc.

### 4.1. Saccharides

#### 4.1.1. Mannose

Mannose is commonly found on the surface of bacteria, fungi, and viruses [58]. A variety of immune receptors can recognize mannose, including different CLRs (e.g., mannose-binding lectin, mannose receptors, Mincle [59], and DC-SIGN receptors [21]) and TLR4, which binds O-linked mannosylated ligands [60]. Binding can induce complement activation and phagocytosis [5,11], triggering innate immune responses [61]. Furthermore, stimulation of these receptors can also induce receptor-mediated endocytosis and influence TLR signaling cascades, activating the adaptive immune system [42,62,63]. For example, mannose receptors CD206 and CD209, which are C-type lectins expressed on macrophages and DCs, recognized mannosylated antigen-bearing constructs and delivered antigens onto MHC I and MHC II receptors. These were further recognized by T-cells, triggering adaptive immunity [64]. Activation of mannose receptors enhances CD4^+^ and CD8^+^ T-cell responses, inducing Th1 and Th2-type immunity, which in turn induces IgG production and long-lasting immunity [62,63,65]. The activation of mannose receptors in tumor-associated macrophages has also been shown to improve innate and adaptive antitumor immunity [11]. Therefore, mannose is often used in vaccine design to enhance the immunogenicity of co-administered antigens.

Glaffig et al. developed anticancer vaccines where two mannosyl glycolic acids were linked to both amino groups of the N-terminal lysine of a peptide derived from mucin 1 protein (which is overexpressed by several cancers) [66]. The mannosylated antigens were internalized via endocytosis by DCs and macrophages 2–4 orders of magnitude faster than non-mannosylated vaccine candidates. The mannosylated compounds also triggered an increase in the number of macrophages, DCs, and CD4^+^ T-cells in the local lymph organs of mice. The vaccine induced the production of high titers of IgG specific to tumor-associated glycopeptide antigens. Apostolopoulos and coworkers reported that mucin 1 conjugated to mannose under oxidizing conditions caused the endosomal escape of antigens into the cytoplasm, presented mucin 1 peptides into the MHC I pathway, generated a Th1 response, and yielded a high level of CTL and a low level of IgG2a antibodies compared to other subtypes [67,68]. The vaccine candidate also increased the level of IL-12 and IFN-γ cytokines. Overall, the mannosylated antigen enhanced cross-presentation, a pathway where tumor-associated antigens can induce cytotoxic CD8^+^ T-cell activation and resulted in induction of stronger antitumor immunity [69].

To enhance the immunogenicity of ovalbumin (OVA), a combination of TLR7 ligand and mannose-targeting moiety was tested [70]. The mixture of imidazoquinolines, TLR7 agonists, and mannose was conjugated to OVA through a self-immolative linker via a metal-free cycloaddition reaction. This model vaccine amplified antigen presentation to T-cells and generated greater humoral and cellular immunity than vaccine candidates lacking either a mannose-receptor targeting moiety or a TLR7 ligand. The same strategy was also used for the delivery of *Plasmodium falciparum-*derived circumsporozoite protein, with the result of high IgG production and the inhibition of sporozoite invasion into hepatocytes in mice.

**Figure 3:**
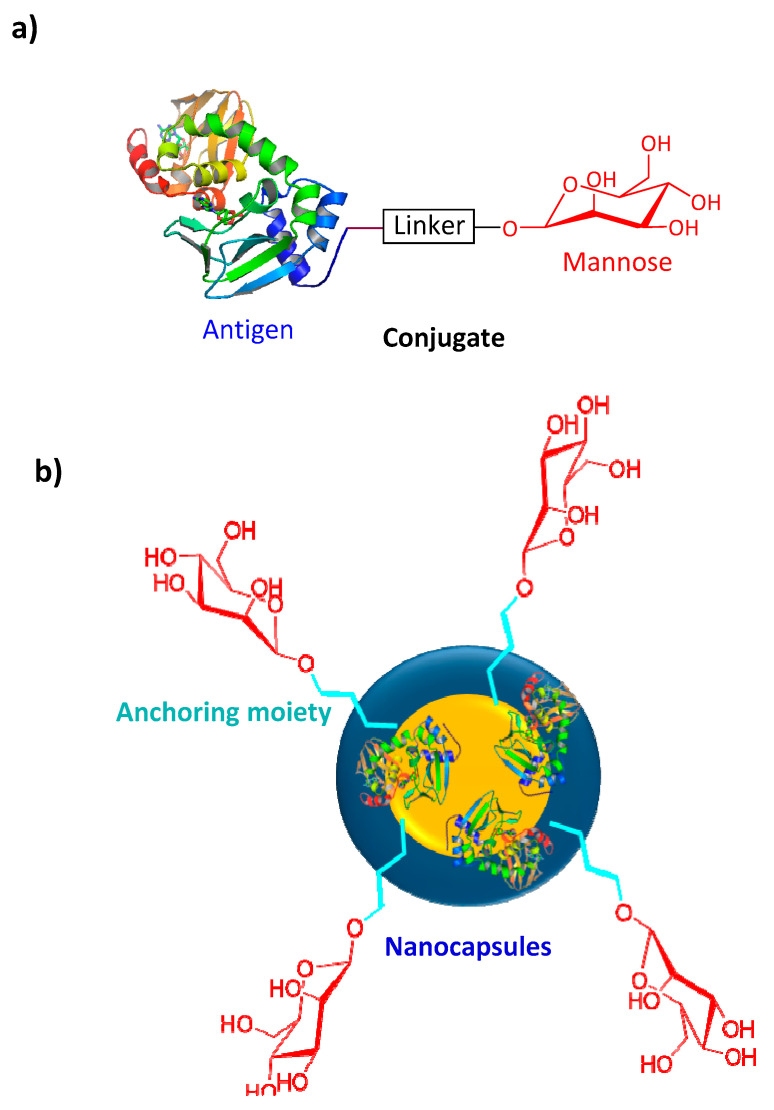
Mannosylated peptide/protein-based vaccine constructs: (**a**) antigenic peptide/protein conjugated to mannose; (**b**) mannose conjugated to a polymer/lipid-based nanocapsule.

Nanoparticles have recently become one of the most popular and effective vaccine delivery systems [71], and mannose has been employed to modify some of these vaccine constructs (Figure 3a,b). Coating the surface of nanoparticles and nanocapsules with mannose has been found to enhance antigen uptake by APCs [72]. For example, alginate was conjugated to OVA (model antigen) via a pH-sensitive Schiff base bond [61]. Tetrabutylammonium-alginate and 4-aminophenyl d-mannopyranoside were also used to synthesize mannosylated alginate. Nanoparticles were then formed by cross-linking two types of modified alginates with the help of CaCl_2_. The nanoparticle size and zeta potential were 310 nm and −46 mV, respectively. These nanoparticles were promptly taken up by mouse bone marrow DCs (BMDCs) *in vitro*. Uptake of mannose-free nanoparticles was 3-fold lower, and subsequent activation of APCs was 2-fold lower (when measured by APC CD40 marker overexpression). Furthermore, cross-presentation of antigen and CTL activation were significantly enhanced when mannosylated nanoparticles were administered to mice. In another study, poly d-l-lactide-co-glycolide nanoparticles were loaded with imiquimod as a TLR7 agonist, coated with cancer cell membrane as an antigen, and mannose was linked to the nanoparticles via a lipid-based anchoring moiety [73]. The mannosylated vaccine candidates enhanced nanoparticle DC uptake and stimulated DC maturation. Compared to mice treated with free nanoparticles, mannosylated nanoparticles effectively migrated to draining lymph nodes and triggered tumor-specific immune responses. The lymph node retention of the nanovaccine was also enhanced with the help of mannose. The treatment of mice with an anti-programmed death-1 (anti-PD-1) checkpoint blockade failed to cure mice in tumor challenge experiments. However, once both strategies, mannosylation and anti-PD-1 blockage, were combined, half of the mice survived the tumor challenge.

The polysaccharide, chitosan, is used widely for vaccine delivery due to its mucoadhesive and adjuvating properties [40,74,75,76,77]. Following subcutaneous administration in mice, nanoparticles (120 nm, −12 mV) formed by electrostatic interactions between chitosan and mannosylated alginate and loaded with tumor cell lysates were efficiently taken up by DCs in the draining lymph node [78]. Moreover, the mannosylated nanoparticles enhanced the expression of APC maturation markers, including MHC I and MHC II, CD40, CD80, and CD86, and stimulated higher TNF-α and IL-12 levels in serum. The activity of CTLs was analyzed using the CD107 marker of CTL degranulation and the level of IFN-γ expression. This was significantly higher in the lymph node and spleen following the administration of mannosylated nanoparticles, in comparison to non-mannosylated nanoparticles and free antigens. The nanoparticles delayed the growth of early-stage tumor cells; however, when mice were challenged with 4T1 mouse breast tumor cells and then immunized, mannosylated vaccine candidates failed to provide antitumor activity.

Liposomes are another popular vaccine delivery strategy [79], and carbohydrates have similarly been utilized within these formulations. Mannose can be easily anchored to liposomal surfaces by lipidation. For example, mannosylated lipidated peptide was anchored to the surface of liposomes prepared by lipid film hydration [80]. The mannosylated liposomes efficiently increased antigen uptake by APCs and up-regulated expression of MHC II and co-stimulatory molecules, including CD80 and CD86 on both DCs and macrophages. In contrast, corresponding non-mannosylated liposomes were poor activators of APCs. Mannose-modified liposomal vaccines can improve vaccine efficacy, not only by inducing and promoting the desired immune response, but also by decreasing adverse effects by minimizing interaction with irrelevant cells and tissues. OVA-loaded 1,2-dioleoyl-3-trimethylammoniumpropane (DOTAP) liposomes and DOTAP-polyethylene glycol-poly caprolactone (PEG)-mannose liposomes have both been studied. The liposomes with mannose showed dynamic lymphatic trafficking *in vivo* with accumulation in draining lymph nodes in a short period, indicating accelerated drainage from the injection site into lymph nodes [81]. Furthermore, the mannosylated liposomes improved cross-presentation and cytokine production, stimulated greater lymphocyte activation, CD4^+^, and CD8^+^ T-cell response, effector cytokine secretion, and induced Th1-biased humoral responses compared to non-mannosylated liposomes [82]. Zhu and coworkers produced hybrid liposomes, named polymersomes [62]. Polycaprolactone (PCL)-PEG-PCL polymersomes bearing 1,2-distearoyl-sn-glycero-3-phosphoethanolamine (DSPE)-PEG-mannose (mannose functionalized lipid hybrid polymersome), which was synthesized by covalent binding with DSPE-PEG-NH_2_ and d-mannopyranosylphenyl isothiocyanate, were formed. In addition, positively charged lipid (DOTAP), TLR4 agonist (lipid A), and TLR7/8 agonists (imiquimod) were incorporated into the hydrophobic layer of polymersomes. The negatively charged model antigen OVA, was encapsulated electrostatically into polymersomes (220 nm and −1.6 mV) and the construct was found to be non-toxic to mouse BMDCs. The polymersomes were taken into BMDC twice as efficiently as OVA alone, and OVA mixed with lipid A. The liposomes were recognized by mannose receptors, facilitating antigen uptake via endocytosis into a distinct endosome subpopulation. The antigens were also protected from lysosomal protease degradation and were allowed to escape into the cytoplasm [83]. Therefore, mannosylated polymersomes effectively cross-presented antigens and produced antigen-specific CTL responses, which were further verified by a mouse tumor challenge with EG7-OVA cells. The mannosylated polymersomes prolonged tumor-free time, effectively suppressed tumor growth, and greatly extended the median survival time of mice [62].

#### 4.1.2. Oligo- and Polysaccharides of Mannose

**Figure 4:**
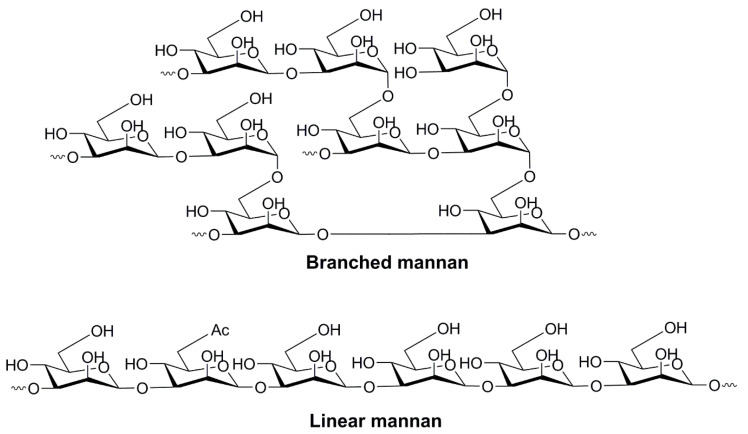
The branched and linear forms of mannan.

The oligo- and polymerized forms of mannose have also been assessed and found to be promptly recognized by PRRs on the surface of human immune cells (e.g., DCs, macrophages, epithelial cells, and endothelial cells) [60]. Mannan, the polymerized form of mannose, can be recognized by a wide range of receptors, including mannose receptors [84], dectin-2 [85], dectin-3 [86], Mincle, DC-SIGN [87,88], galectin-3 [89], FcγR [90], TLR2, TLR4 [91], and TLR6 [92,93]. Therefore, mannan serves as a promising APC-targeting agent that can enhance the uptake and processing of co-administered antigens in APCs. Although it is recognized by the immune system in a similar manner to mannose [92], mannan is a more potent adjuvant. It binds more effectively to receptors consisting of multiple carbohydrate-recognizing domains as it bears multiple ligands (mannose moieties) [94]. Mannan comprises linear and branched polymers of mannose sugars linked via α-1,2, α-1,3, α-1,4, α-1,6, and β-1,2 glycoside bonds (Figure 4) [95]. The ligand–receptor interaction and the stimulation of immunity depends on the conformation of mannan (and other polysaccharides, too), the types of glycosidic bonds in the molecule, and the degree of branching, charge and molecular weight [96]. Interestingly, coating nanoparticles with mannan enhances not only vaccine delivery to APCs, but also prevents nanoparticle aggregation [97].

Nanoparticles chemically modified or coated with oligomannose and polymannose are popularly applied for vaccine delivery [9]. For example, a series of diether lipids were conjugated to mannose (Man), trimannose (Tri-Man), branched trimannose (Man-Tri), tetramannose (Tetra-Man) or pentamannose (Penta-Man) through “click chemistry” between azido mannose oligosaccharide and the diether lipid (propargyl-PEG8-diether) [21]. Mannosylated lipids were anchored to liposomes and their ability to be taken up by mannose receptors or DC-SIGN was analyzed using DC2.4 cells and DC-SIGN HEK293 cells, respectively. Man-Tri was preferentially taken up by DC-SIGN receptors, while linear Tri-Man and Man were recognized preferentially by mannose receptors. In contrast to mannose receptors, which are expressed in a variety of cells, DC-SIGN is expressed predominantly on DCs. Therefore, the branched form of the mannosylated vaccine can be delivered more selectively to DCs. Interestingly, the higher oligomers of mannose (Tetra-Man and Penta-Man) were not taken up by APCs any more efficiently than Tri-Man.

The conjugation strategy used to combine mannan with antigens also plays an important role in receptor recognition. Mannan conjugated to the breast cancer antigen, mucin 1, under either oxidation or reducing conditions, was injected intraperitoneally into mice [68]. The oxidative conjugation led to CTL activation and Th1 cytokine (IFN-γ) production, while stimulation of antibody (predominantly IgG2a) production was poor. In contrast, the reductive coupling between mannan and the antigen stimulated strong humoral immunity and no cellular response. Mice immunized with oxidized mannan coupled with the antigen were protected in a tumor challenge with mucin-1 3T3 tumor cells, whereas tumor growth was not inhibited in mice treated with reduced mannan-antigen conjugate. It has been suggested that the oxidizing conditions allowed mannan aldehyde groups to form Schiff bases not only with antigens, but also with APCs, inducing strong antitumor immunity by targeting the antigen to the intracellular processing pathway for presentation with MHC I molecules. However, this was contradicted by another study that showed high antibody responses against vaccines bearing oxidized mannan [98]. A nanoparticle-based vaccine candidate (200–800 nm) against porcine circovirus type 2 (PCV2) containing oxidized mannan attached to PCV2_ΔCap42-233_ protein via an acid-sensitive Schiff base reaction was developed. When subcutaneously administered, it showed a higher level of IL-4 (which regulates antibody-mediated humoral immune responses), and consequently higher antibody production, than non-mannanylated vaccine candidates. The level of IFN-γ was not significantly higher in mice treated with a mannan-bearing vaccine. The conflicting outcome of the two studies can be related to different abilities of the vaccines to form nanoparticles, and/or the varied structural nature (branching, molecular weight, even purity) of the mannans used. Thus, despite the mannan’s advantage of being able to stimulate stronger immune responses, the systems are less defined and show greater variability in immune responses than mannose.

The immune-stimulating ability of reductive and oxidative mannan-antigen conjugates can also be affected by the route of administration. For example, mannan was coupled to secreted listeriolysin O (LLO) protein, the immunodominant antigen of *Listeria monocytogenes,* or to the 19-kDa protein (19-FP) secreted by *Mycobacterium tuberculosis* under either oxidation or reductive conditions. These were each administered intranasally or intraperitoneally [99]. The intranasally administered oxidative mannan-LLO conjugate induced greater humoral immune responses, especially LLO-specific IgA, IgG1, and IgG2a titers, in comparison to the conjugate-administered intraperitoneally. IgA antibody titers from vaginal washings induced by LLO- or 19-FP-mannan conjugates administered through the intranasal route were greater than those induced by a physical mixture of mannan and LLO or 19-FP. Oxidized mannan-LLO induced significantly higher LLO-specific IgA, IgG1, and IgG2 antibodies than the reduced mannosylated antigen. This suggested that the mucosal adjuvanticity of mannan and the induced immunity of both oxidized and reductive mannan-antigen conjugations were administration route-dependent.

#### 4.1.3. Glucan

Glucan, the polymerized form of glucose and a natural component of the cell wall of yeast and some bacteria, has been found to have immune-stimulating activity [100]. The polysaccharide is linked by a variety of glycosidic bonds, such as α and β-1,3, and 1,4 glycosides bonds. Moreover, glucan can be isolated (e.g., from *Saccharomyces cerevisiae*) as hollow spherical particles (GPs) [101]. All of the homopolymer forms of glucose are recognized by immune cells (e.g., neutrophils, macrophages, and DCs) [102]. Glucan binds to various PRRs, such as dectin-1, TLR2, TLR6, and TLR9, and therefore stimulates phagocytosis and endocytosis of antigens leading to a proliferation of CD4^+^ and CD8^+^ cells, differentiation of Th1 and Th17, the up-regulation of cytokines IL-4 and IL-3, and the production of high levels of IgG and IgA antibodies [56,60,103,104]. Furthermore, glucan is non-toxic and has anticoagulant, antithrombotic, and antioxidant properties [105]. Glucan and related GPs have been used for vaccine delivery. For example, β-glucan was conjugated to three hollow silica particles, which were produced on the following templates: (a) *Escherichia coli* particles (rod, 900 nm × 1.2–3.2 µm), (b) *Staphylococcus aureus* particles (spherical, 900 nm), and (c) polystyrene (spherical, 220 nm) [56]. The particles were then loaded with OVA. All glucan-conjugated particles were taken up by dectin-1, complement receptors, and TLR-2 on APCs. This successfully induced APC maturation, overexpression of MHC II, and strong IgG antibody responses in comparison to plain particles. All glucan particles induced Th1 and Th2 responses with only a minor difference in Th1/Th2 specificity between particles.

The high solubility of GPs and their strong capacity to entrap antigenic protein and peptides into the inner hollow cavity of the particles make GPs interesting vaccine delivery vehicles. In addition, owing to their ability to interact with different human epithelial cell lines, glucan-based delivery systems can enhance mucosal immunity and elicit humoral responses. For example, GPs were loaded with OVA by dissolving OVA in hydrated GPs followed by lyophilization (particle size = 3.7 μm, zeta potential = −6.5 mV, and entrapment efficacy = 98%) [106]. OVA-loaded GPs enhanced the expression of dectin-1, TLR2, proinflammatory cytokines, and chemokines. GPs were recognized and internalized by M-cells in PPs. Finally, the GPs promoted Th17 responses and OVA-specific IgA production; however, they did not trigger higher IgG production than OVA alone following oral vaccination in mice.

Various types of glucan homopolymers exist in nature, and their biological and immunological properties depend on their molecular and structural features, such as polymer length, branching degree, type of glycosidic bond, and solubility. The immune-stimulating properties of different glucans were reviewed by Moreno-Mendieta et al. [45]. Curdlan sulfate (β-1,3 glucan) recognized by dectin-1, cellulose (β-1,4-glucan), lentinan (β-1,3-linked glucan with a single β-1,6 branch every 5 residues) recognized by TLR4, and phycarine or laminarin (β-1,3-linked glucan with a single β-1,6 branch) binding to complement receptor 3, induced activation of different immune pathways. For example, sparan, a six-branched β-1,3 glucan obtained from the medicinal mushroom *Sparassis crispa,* was recognized through TLR4 and led to the activation of mitogen-activated protein kinase and NF-κB signaling pathways [107]. It induced DC maturation as measured by the overexpression of MHC I and II and co-stimulatory molecules (CD40, CD80, and CD86). Additionally, sparan enhanced the production of IL-12, IL-1, TNF-α, IFN-γ, and IL-2.

Glucan-rich bacterial extracts are also used as adjuvants. Zymosan is an insoluble cell wall extract from *Saccharomyces cerevisiae* that is composed of glucan (55%), mannan, proteins, lipids, and chitin [108] and it is recognized by dectin-1 and TLR2 [109,110]. Co-activation of dectin-1 and TLR2-signaling pathways, mediated by zymosan, results in a synergistic increase in the production of IL-12 and TNF-α. Influenza vaccines adjuvanted with polyinosinic:polycytidylic acid (poly (I:C)), zymosan, and A/PR8 antigens have been examined in BALB/c mice [111]. Poly (I:C) induced TLR3-mediated signaling pathways and zymosan induced dectin-1 and TLR-2-mediated signaling pathways in BMDCs. Secretory IgA and serum IgG levels were increased significantly in mice immunized with the vaccine candidates carrying both adjuvants (zymosan and poly I:C) compared to those with vaccine candidates bearing a single adjuvant. In addition, mice immunized with a vaccine carrying both adjuvants (zymosan and poly I:C) developed a protective response to the influenza virus, and had an increased survival rate and a reduced weight loss.

#### 4.1.4. Chitosan

**Figure 5:**
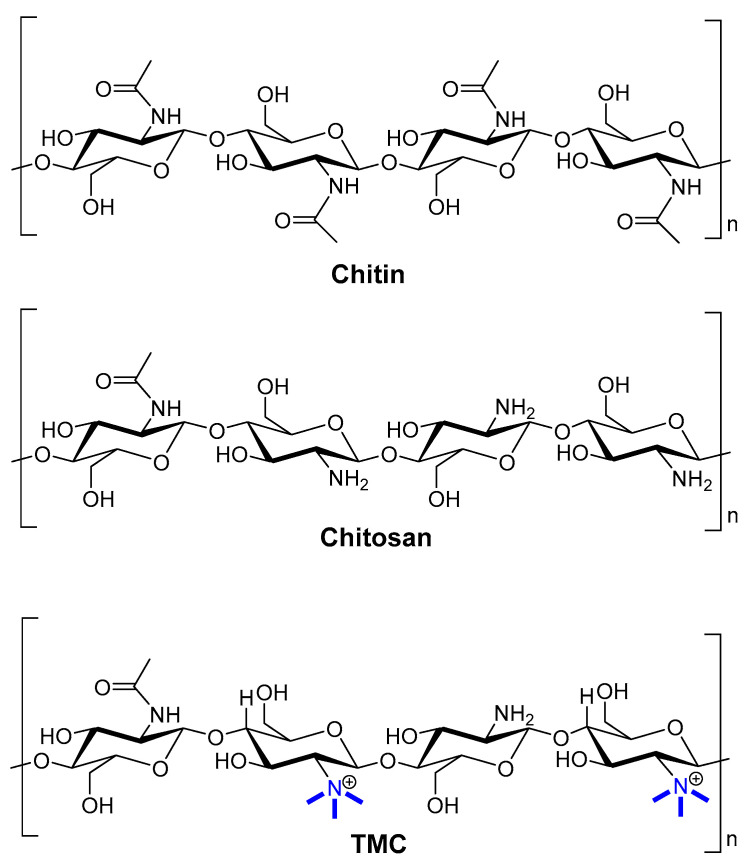
Comparison of the chemical structures of chitin, chitosan, and TMC.

Chitosan is a linear amino polysaccharide composed of β-1,4-linked monomers of d-glucosamine and *N*-acetyl-d-glucosamine (Figure 5). It is usually produced by deacetylation of chitin (β-1,4-*N*-acetyl-d-glucosamine) isolated from animals (e.g., crayfish, shrimp waste, crabs, and lobsters) or fungal resources (e.g., *Agaricus*, *Hydnum*, and *Boletus* species) [13,112]. However, the low solubility of chitosan hinders its applicability to drug and vaccine delivery. To increase its solubility and stability, chitosan is often chemically modified to produce glycated chitosan, thiolated chitosan, monomethyl chitosan, and trimethyl chitosan (TMC) [76,95]. TMC bearing a permanent positive charge (Figure 5) is the most hydrophilic compound among these derivatives. Moreover, TMC can be easily produced in a cost-effective manner. It has high antimicrobial activity, high absorption through biological membranes, and it is mucoadhesive and non-toxic [113]. Furthermore, TMC has immune-stimulating properties, which typically exceed those of anionic polysaccharides [114]. Its mucoadhesive properties retain vaccine formulations on mucosal surfaces, so chitosan/TMC is often used for intranasal vaccine delivery [115]. Chitosan/TMC can also open the tight junctions between epithelial cells, further improving vaccine uptake [116]. Consequently, chitosan/TMC-based nanoparticles easily reach M-cells [115], penetrate epithelial cells, deliver antigens to immune competent cells, and induce both systemic and mucosal immunities [117]. For example, a lipopeptide-based vaccine bearing J14 peptide epitopes derived from group A *Streptococcus* (GAS) M-protein was loaded into polymeric nanoparticles. These nanoparticles (100–300 nm) were prepared from dextran, poly d,l-lactic-acid-co-glycolic acid (PLGA), and/or TMC via a two-stage double centrifugation technique [114]. Negatively charged polymers (PLGA and dextran) were used to formulate polyelectrolyte nanoparticles with the positively charged lipopeptide [118]. Dextran and dextran/TMC nanoparticles were both efficiently taken up by DCs, but only dextran/TMC significantly enhanced the maturation of DCs, as measured by overexpression of all tested co-stimulatory molecules (i.e., CD40, CD80, and CD86). Dextran/TMC nanoparticles were also the most effective in the induction of antigen-specific systemic (IgG) and mucosal (IgA) antibody titers following intranasal immunization, and produced antibodies showing a high opsonic activity against tested clinical GAS isolates *in vitro*. In other strategies, TMC was used to form nanoparticles (~200 nm, +36 mV) with GAS peptide epitope-polyglutamic acid (PGA) conjugates via electrostatic interaction between PGA and TMC [119]. These nanoparticles induced DCs and macrophage maturation (overexpression of MHC II and CD80); while either PGA or TMC mixed with antigens did not. Moreover, the nanoparticles induced significantly higher IgG and IgA antibody titers than antigens adjuvanted with cholera toxin B subunit (CTB). Mice immunized with the nanoparticles showed a reduced bacterial load when challenged with M1 GAS, while the CTB-adjuvanted vaccine did not. This system was further improved by antigen lipidation and the replacement of crustacean TMC with better-defined, low molecular weight, highly deacetylated fungi-derived TMC [120,121].

In addition to its mucoadhesive properties, chitosan also acts as a receptor ligand and binds to NLRs, dectin-1, leukotriene B4 receptor [122], and TLR2, TLR4, and TLR5 on APCs [123]. Moreover, the glucosamine units in chitosan are recognized by mannose receptors [124]. Chitosan activates macrophages [13,125], induces cytokine secretion from NK cells, and stimulates NALT to produce mucosal secretory IgA, IgG, TNF-α, IL-6, and IFN-γ [43]. PCV2 subunit vaccine based on the PCV2 capsid protein was conjugated with chitosan oligosaccharide and OVA-based carriers [123]. The chitosan in the vaccine formulation increased cell proliferation, phagocytosis activity in macrophages, TLR2 and TLR4 expression on macrophages, and subsequently increased antigen presentation. Moreover, the vaccine candidate consisting of chitosan conjugated with PCV2 increased RAW 264.7 macrophage proliferation, nitric oxide production (playing a critical role in the activation of immune cells, such as macrophages), and proinflammatory cytokine production. It also significantly increased the levels of TNF-α, IFN-γ, IL-1β, IL-6, and IL-8 in comparison to PCV2 alone, protein-based carrier loaded PCV2, and chitosan-mixed PCV2.

Chitosan and TMC particles can disrupt lysosomes using the “lysozyme escape pathway”. Antigen presentation is then possible through the MHCI pathway, which primes CD8^+^ and evokes strong CTL responses [126]. OVA has been entrapped in nanosheets formed by catechol-modified chitosan/calcium phosphate via coprecipitation (thickness = 200–300 nm, width = 5–20 μm) [127]. The *in vitro* cross-presentation of OVA by BMDCs was evaluated by a LacZ antigen presentation assay. The antigen uptake, antigen endo/lysosomal escape, and antigen cross-presentation was facilitated by the nanosheets and they enhanced the expression of MHC I complexes and CD8^+^ T-cell activation.

#### 4.1.5. Hyaluronic Acid

**Figure 6:**
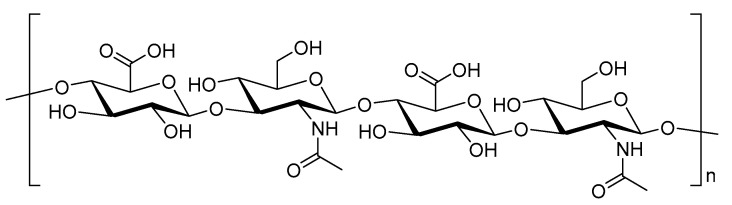
The chemical structure of hyaluronic acid.

Hyaluronic acid (HA), known as hyaluronan, is a linear anionic and hydrophilic polysaccharide based on (β-1,4 and β-1,3) d*-*glucuronic acid and *N-*acetyl*-*d*-*glucosamine [128] (Figure 6). This polymer is produced via extraction from rooster combs and through bacterial fermentation. It is recognized by a variety of receptors, including the receptor for hyaluronic acid-mediated motility (RHAMM) [129], transmembrane protein layilin, hyaluronic acid receptor for endocytosis (HARE), lymphatic vessel endocytic receptors (LYVE-1) [130], and intracellular HA-binding proteins, including CDC37, RHAMM/IHABP, P-32, and IHABP4 [131]. Moreover, it interacts with dermal DCs and epidermal Langerhans cells through HA receptors, TLR2, and TLR4 present in immune cells [132]. HA receptors are expressed in keratinocytes, which are the most abundant cells in the epidermis, and fibroblasts in the dermis. Upon activation by low molecular HA, keratinocytes produce an innate immune response (release β-defensins) [133].

HA has been explored as a potential adjuvant for antigen delivery due to its immune-stimulating properties and its ability to form nanoparticles via electrostatic interaction with cationic components (cationic polymers, or some lipidic formulations). HA-coated nanoparticles are able to promote DC activation and maturation, enhance co-stimulatory molecules such as CD40 and CD86, induce antigen-specific CD4^+^ and CD8^+^ T-cell responses, stimulate the secretion of cytokines, facilitate robust antigen-specific IgG antibody production, and enhance memory T-cell generation [129,134]. Nanoparticles were formed by ionic interaction between TMC and HA (321 nm, +13 mV). Thiolated TMC and thiolated HA also formed nanoparticles (TMC-S-S-HA) via ionic gelation followed by spontaneous disulfide formation (338 nm, +7 mV). A third type of nanoparticles was formed by the conjugation of maleimide PEG to the remaining thiol groups of TMC-S-S-HA (352 nm, +4 mV) [135]. OVA was loaded into the nanoparticles and these vaccine candidates were administered by nasal and transdermal routes in mice. The non-PEG (TMC-S-S-HA) nanoparticles induced significantly higher OVA-specific IgG antibody titers compared to PEGlyated TMC-S-S-HA upon nasal administration. However, plain OVA induced higher OVA-specific IgG titers than the mixed TMC/HA nanoparticles. In addition, mice immunized transdermally with TMC-S-S-HA vaccine candidates (either with or without PEG) and mixed TMC/HA induced significantly higher OVA-specific IgG antibody titers than plain OVA. All three nanoparticle compounds induced higher IgG1 titers than IgG2 titers; however, TMC-S-S-HA (either with or without PEG) elicited significantly higher IgG1 titers than mixed TMC/HA nanoparticles.

HA-coated cationic nanoparticles can be taken up by APCs via HA-CD44 receptor-mediated endocytosis, and at the same time undergo an endosomal escape through the hyaluronidases-catalyzed degradation of HA in the endosome or lysosome [136]. For example, DOTAP/PLGA hybrid nanoparticles encapsulating OVA were coated with HA (HA-DOTAP-PLGA) (236 nm, +22 mV) [134]. HA-DOTAP-PLGA nanoparticles were taken up by HA-CD44 receptor-mediated endocytosis, and OVA encapsulated in the HA-DOTAP-PLGA partially escaped from the lysosomes into the cytosolic space. Thus, the antigen was presented by both MHC I and MHC II pathways. The level of co-stimulatory molecules CD40 and CD86 were also increased significantly in mice immunized with HA-DOTAP-PLGA. Moreover, Th1 polarizing cytokine (TNF-α and IL-12) and Th2 polarizing cytokine (IL-6) levels were notably higher in mice immunized with HA-DOTAP-PLGA in comparison to those injected with nanoparticles lacking HA or free OVA. HA-DOTAP-PLGA enhanced the cellular uptake efficiency, cytoplasmic delivery of antigens in BMDCs, and promoted DC activation and maturation. Mice immunized with HA-DOTAP-PLGA also stimulated high OVA-specific CD4^+^ and CD8^+^ T-cell responses and induced balanced Th1/Th2 responses, with higher IgG levels, and memory T-cell responses.

To improve HA’s stability, hydrophobicity, and immune-stimulatory potential, it has been modified into an ester form, known as HYAFF^®^ [137]. HYAFF microspheres prepared using coacervation phase-separation and loaded with a non-toxic mutant of *Escherichia coli* as a mucosal adjuvant (LTK63) [138] and hemagglutinin (influenza antigen) (32 µm) were produced and injected intranasally into micro-pigs (8–10 kg); antigen alone was applied intramuscularly. The animals intranasally immunized with HYAFF microspheres produced higher IgG and IgA antibody titers against the antigen than those vaccinated with antigen with LTK63 or the antigen alone administered intramuscularly [139].

### 4.2. Saccharide Derivatives

#### 4.2.1. Lipid A and its Derivatives

**Figure 7:**
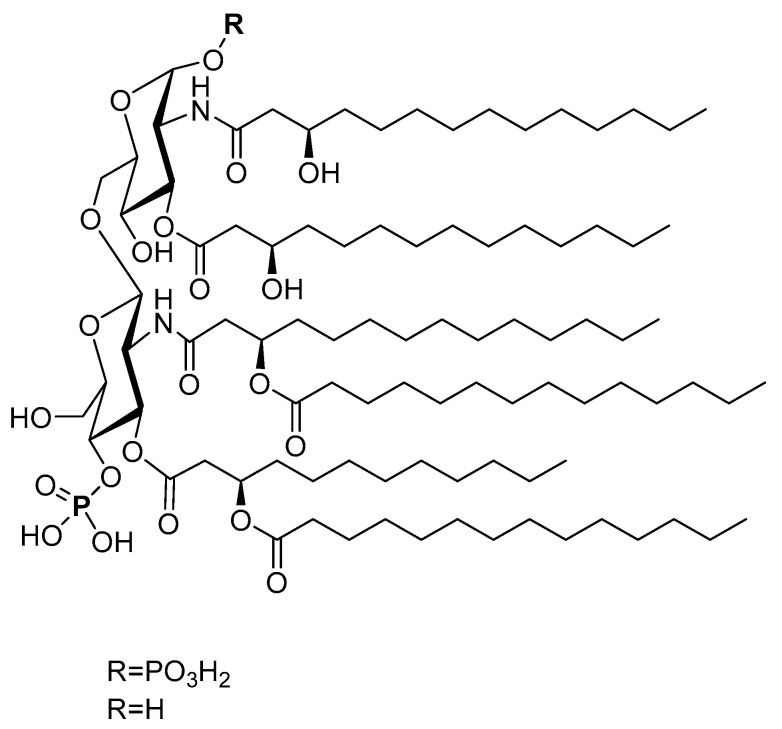
An exemplary structure of lipid A (R = PO_3_OH_2_) and MPLA (R = H).

Besides basic monosaccharides and their polymers, carbohydrate derivatives (e.g., lipidated carbohydrates) can also serve as APC receptor ligands and act as adjuvants. Lipid A is a lipid component of a bacterial endotoxin. Its structure varies slightly between different Gram-negative bacteria species, such as *Acinetobacter baumannii*, *Burkholderia pseudomallei*, *Campylobacter jejuni*, *Escherichia coli*, *Helicobacter pylori*, *Klebsiella pneumoniae*, *Neisseria gonorrheae*, and *Salmonella Minnesota* R595 [140,141]. In general, lipid A usually contains a disaccharide (two glucosamine residues), two phosphate groups, and five or six fatty acids, with a chain length of 12 to 16 carbon atoms (Figure 7). Even when isolated from a single microorganism, lipid A is usually a mixture of several compounds that differ in fatty acid composition. It is one of the strongest naturally sourced adjuvants available; however, it is also very toxic [142]. Advantageously, the lipid A derivative, monophosphoryl lipid A, which lacks one phosphate group (MPLA, Figure 7), has reduced toxicity but still has a strong immune-stimulating ability.

MPLA is recognized by TLR4 on the plasma membrane of APCs [24]. Upon recognition, it enhances the activation of APCs, triggers the expression of pro-inflammatory (IL-1, IL-6, TNF-α) and anti-inflammatory (IL-10) cytokines, and induces antigen-specific Th1, Th2, and Th17 responses and the production of antigen-specific antibodies [143]. It can also transport antigens into the cytoplasm, present endogenous antigens through MHC I molecules, and activate CD8^+^ T-cell immune responses [144]. MPLA enables the induction of antitumor immunity via cross-presentation of the antigen. Therefore, both humoral and cellular responses against infectious diseases, such as influenza [145] and cancer, could be induced by MPLA-based formulations.

MPLA-adjuvanted vaccines have been tested against a wide variety of diseases, including influenza [146], hepatitis B [147], rabies [148], and parasitic infections [149], among others. The MPLA vaccine for rabies promoted the induction of stronger cell-mediated immune responses, including the production of IL-4, IFN-γ and the activation of CD4^+^/CD8^+^ T-cells compared to formulations without MPLA [148]. *Toxoplasma* GRA2 and GRA6 antigens mixed with MPLA induced a strong Th1 response and a significant increase in both IFN-γ and IL-2 expression in mice [149]. Similarly, PLGA nanoparticles (300 nm) loaded with viral nucleocapsid hepatitis B core antigen (HBcAg) and MPLA also induced a mainly Th1 immunity [147].

In addition to the capacity of MPLA to induce and enhance systemic immunity, it can also serve as a mucosal adjuvant. Influenza antigens (Flu Shield) were co-administered with MPLA through intranasal and subcutaneous routes [150]. Intranasally immunized mice produced higher levels of antigen-specific systemic (IgG1 and IgG2) and mucosal antibodies (IgA) at the site of administration and distant mucosal sites than non-MPLA vaccine formulations. High levels of IgG2a serum antibodies were produced, consistent with the release of the Th1 cytokine, IFN-γ. The mice were fully protected from the lethal influenza (A/HK/68) challenge; however substantial weight loss (6.5 g) was observed. The formulation was less effective overall when administered subcutaneously (survival = 78%; weigh loss = 8.8 g), and only systemic immune responses were produced; no IgA production was detected. Mice treated without MPLA had a lower survival rate (22%) and a greatly increased weight loss (11.2 g). In summary, MPLA promoted not only systemic and mucosal responses following intranasal immunization, but also protected mice challenged with the virus.

Consequently, a variety of vaccines adjuvanted with MPLA have been developed. Several subcutaneously delivered MPLA vaccines have been approved for commercial use, for example Pollinex^®^ Quattro (against pollen allergies) [151] and Fendrix (against hepatitis B) [152]. Intramuscularly administered MPLA vaccines against *Plasmodium falciparum* [153], *Neisseria meningitidis* [154], and *Herpes simplex* [155] have been tested in clinical settings.

MPLA has also been used in combination with other adjuvants. For example, both MPLA and TLR9 agonists (CpG-containing oligodeoxynucleotide) were used for the development of a vaccine against respiratory syncytial virus (RSV). Fusion protein (purified RSV A2 F glycoprotein) adjuvanted with CPG + MPLA triggered higher IgG antibodies titers in intramuscularly immunized infant and adult mice compared to antigen adjuvanted with just MPLA and CPG [156]. The levels of antigen-specific IgG1 and Th2 cytokines (IL-4, IL-5, and IL-13) were relatively low, whereas the levels of IgG2 and Th1 cytokine (IFN-γ) were higher in mice immunized with CPG + MPLA compared to mice treated with single adjuvant-based formulations. When challenged with RSV, CPG + MPLA-bearing vaccine cleared lung viral loads most effectively, followed by alum, CpG, and MPLA-adjuvanted formulations. MPLA-based adjuvant combinations have also been tested against malaria. *Plasmodium falciparum* cell-traversal protein was formulated with three different vaccine adjuvants, MPLA, CpG, and saponin from the tree *Quillaja saponaria* (QS-21) and analyzed in BALB/c mice [157]. Combinations of the three adjuvants and CPG alone produced higher levels of IgG than MPLA. However, the IgG antibody level decreased after the first boost in all adjuvanted groups, and the highest reduction was observed in the CPG group. The amount of TNF-α and IFN-γ were significantly higher in mice treated with the complex adjuvants compared to vaccine constructs bearing a single adjuvant. The effect of mouse polyclonal antibodies on the inhibition of *P. falciparum* NF54 infection of the female *Anopheles stephensi* mosquito was assessed using a standard membrane feeding assay. Antibodies isolated from mice immunized with the complex adjuvant-based vaccine showed the highest inhibition of oocyst intensity (88%) and also the lowest infection prevalence in *A. stephensi* (73%).

Following such observations, GlaxoSmithKline developed and licensed several combination adjuvants, namely: AS01 (liposome + MPLA + QS-21) [158], AS02 (oil in water emulsion + MPLA + QS-21), AS04 (MPLA + alum hydroxide), and AS15 (MPLA + QS-21 + CPG + liposome) [159]. These adjuvants have been used against human papillomaviruses and malaria in clinical trials [160,161].

As a lipidic compound, MPLA can be easily incorporated into liposomal membranes. Liposomal formulation greatly reduced MPLA toxicity while maintaining its adjuvanting ability [162]. For instance, nanoliposomes (120–140 nm, PDI < 0.2) containing CTL peptide epitope derived from tumor-overexpressed rat HER2/neu protein (P5), a universal CD4^+^ T-helper cell epitope (PADRE), and MPLA were examined as a vaccine against breast cancer [163]. To improve peptide incorporation into liposomes, the P5 and PADRE peptides were separately linked to maleimide-PEG2000 DSPE through covalent binding of the thiol group of peptide cysteine. Mice vaccinated subcutaneously with the vaccine (L5PM) induced an effective cellular immune response with significantly higher production of IFN-γ, and stronger CD8^+^ T-cell immune responses than free P5, P5 with PADRE, or empty liposomes. Tumor growth was significantly reduced, and survival time increased in mice immunized with MPLA-adjuvanted liposomes in comparison to liposomes lacking MPLA. In addition to the prophylactic effects of this vaccine formulation, the liposomes had higher therapeutic efficacy compared to non-MPLA liposomal formulations when mice were challenged with TUBO cells and then subcutaneously vaccinated with liposomal vaccine candidates. The same liposomal delivery system incorporating the pH-sensitive lipid, dioleoylphosphatidylethanolamine (DOPE), loaded with P5 and MPLA (133 nm, PDI = 0.176, and −45 mV) triggered a higher level of IFN-γ production, stronger CTL immune responses against the antigen, and more efficient protection when challenged with tumors compared to delivery systems without DOPE and MPLA [164]. However, neither study evaluated whether the liposomal vehicles were the essential component of the vaccine formulation. MPLA co-administered with the antigen was also not tested.

Liposomes containing saturated phospholipids, cholesterol, and MPLA were manufactured as vaccine adjuvants at the Walter Reed Army Institute of Research and referred to as Army Liposome Formulation (ALF) [165]. ALF and ALF-bearing QS-21 (ALFQ) were combined with polysaccharide capsules isolated from *Campylobacter jejuni* strain_81–176_ (type HS23/36) conjugated to CRM_197_ (a non-toxic mutant of diphtheria toxin). These were used as a vaccine against *C. jejuni* [166]. Both vaccine candidates induced higher antigen-specific IgG responses (Th1-mediated IgG2b, IgG2c) than the antigen alone following intramuscular vaccination in mice. ALFQ induced significantly higher production of Th1 cytokines (TNF-α, IFN-γ, IL-2), IL17 and Th2 cytokines (IL-4, IL-10), and antigen-specific IgA responses, and was more effective in a *C. jejuni* challenge compare to the antigen alone or the ALF-adjuvanted vaccine candidate. Torres et al. used ALF liposomes in the formulation of a vaccine against both human immunodeficiency virus 1 (HIV-1) infection and heroin addiction [167]. The linear and branched lipidated V2 loop peptide from the A/E strain of HIV-1 gp120 protein was incorporated into the bilayer of ALF liposomes. The liposomes were mixed with heroin hapten conjugated to tetanus toxoid as a carrier. Upon subcutaneous immunization of mice, the vaccine induced high IgG titers and the produced antibodies inhibited the binding of the V2 loop peptide to the human α_4_β_7_ integrin receptor on CD4^+^ T-cells. The immunized mice also produced high antibody titers against heroin hapten.

MPLA liposomal systems have been tested in comparison to formulations bearing other adjuvants, including Pm_3_CySK_4_ (ligand of TLR2), poly I:C (ligand of TLR3), and R848 (an imidazoquinoline-based ligand of TLR7/8). The adjuvants were incorporated into glycan-coated liposome and loaded with the melanoma tumor antigenic peptide, gp100_280–288_ [168]. Among all tested formulations, the MPLA-adjuvanted vaccine candidate induced notable DC activation with increased expression of the maturation markers CD83 and CD86. Liposome loaded with MPLA was also more efficient than antigen co-administered with soluble MPLA in the induction of antigen cross-presentation. The MPLA liposomal system has been examined in several phase I and II human clinical trials since 1992 [169], including against malaria [170].

Finally, the immune-modulating properties of MPLA can be enhanced through direct conjugation to antigens. For example, MPLA was chemically conjugated to OVA using a carbonyldiimidzole linker to generate stable carbamate-linked OVA-MPLA [7]. The conjugate induced strong CD4^+^ T-cell stimulation and DC activation in comparison to an MPLA/OVA mixture. OVA-MPLA also induced higher pro-inflammatory cytokine responses (IL-1β, IL-6, and TNF-α), anti-inflammatory cytokine responses (IL-10), and antigen-specific Th1 (IFN-α), Th2 (IL-5, IL-13), and Th17 (IL-17) cytokine secretions than either a physical mixture of OVA and MPLA, or OVA or MPLA alone.

Overall, MPLA is a very effective adjuvant for the induction of innate, adaptive (humoral, cellular), and mucosal immune responses. MPLA not only induces DC maturation but also enhances cross-presentation of antigens, and, therefore, can be effectively used in cancer vaccines. While it is already less toxic than lipid A, its toxicity can be further reduced through incorporation into liposomes.

#### 4.2.2. CAF01

CAF01 is an adjuvanted liposomal formulation similar to ALF. However, besides containing a cationic liposome vehicle (dimethyl ioctadecyl ammonium, DDA), it also carries a glycolipid immune stimulator, trehalose-6,6-dibehenate (TDB), a conjugate compromising trehalose and two long acyl chains instead of MPLA [53,55]. In contrast to MPLA, TDB is recognized by CLRs rather than TLR4, and, therefore, the sugar moiety plays a crucial role in its immune recognition. The proposed recognition site for the lipid chains of TDB is the major hydrophobic pocket of the Mincle receptor (a type of CLR). Thus, the lipid moieties also play a role in the formulation’s immune stimulating activity [171]. The presence of two fatty acids (5–14 carbons) is crucial for TDB potency [172].

Various CAF01 vaccine formulations have been tested. Mice subcutaneously immunized with vaccine formulation including epitopes from HIV-1 proteins (e.g., Vif, Gag, Env, Pol, and Vpu), universal T-helper PADRE, and CAF01 [173] induced cellular immune responses against HIV-1 CTL epitopes comparable to incomplete Freund’s adjuvant. When CAF01 was applied as an adjuvant for the tuberculosis vaccine known as Hybrid 1 (H1), carrying the hybrid protein of Early Secretory Antigenic Target (ESAT) and Antigen 85B, it induced a strong and long-lasting (3 years) CD4^+^ T-cell response in a dose-dependent manner [53]. A modified version of the vaccine containing Rv2660 protein (H56), ESAT-6-Ag85B, and CAF01 activated CD4^+^ cells protected mice in a tuberculosis challenge performed one-year post-vaccination [174]. CAF01 was also able to stimulate humoral immune responses. Trivalent influenza split-virion (TIV) vaccine was co-administered with CAF01 into ferrets [175]. The TIV-CAF01-vaccinated ferrets produced significantly higher influenza-specific IgG antibody titers than non-adjuvanted vaccine candidates. The vaccine also reduced the viral load in animals after homologous challenge with A/Brisbane/59/2007 H1N1 influenza.

TDB is recognized by mucosal receptors. It binds to the mucosal homing marker, chemokine receptor-6 (CCR6), and induces strong Th17 and antigen-specific IgA immune responses [176,177]. A mixture of Hirep1 and CTH93 proteins used as *Chlamydia trachomatis* antigens were co-administered with CAF01 through three immunization strategies: subcutaneous; intranasal; or subcutaneous with intranasal boost [178]. Nasal immunization induced weak mucosal and systemic immune responses. Subcutaneous immunization induced the production of high IgG titers but not IgA responses. However, the combination of both delivery routes was effective in inducing strong systemic and mucosal immune responses, indicated by high IgA titers and activation of Th1/Th17, B-, and CD4^+^ T-cells in the lung parenchyma. A similarly designed vaccine against *C. trachomatis* co-administered with CAF01 also induced strong cellular and humoral immune responses and protected mice in *C. trachomatis* SvF challenge [179] when delivered via both subcutaneous and intranasal routes. This was consistent with another study, where parenteral (intramuscular) priming and airway intrapulmonary boost immunization were used for a vaccine formulation including H56 antigens and CAF01 [180]. The immunization strategy of intramuscular vaccination followed by an intrapulmonary boost induced higher lung and systemic H56-specific memory CD4^+^ T-cell activation, IgA and IgG antibody responses, and dose-dependent IFN-γ cytokine responses than the same vaccine delivered via a single administration route.

Additional adjuvants can also be entrapped into CAF01. For instance, a TLR3 ligand, poly (I:C) was incorporated into CAF01 and named CAF05 [181]. It triggered a similar level of antibody production and induced stronger CD8^+^ T-cell responses than CAF01 against incorporated Gag p24 protein [182]. CAF01 was also modified with MPLA and named CAF06 [183]. When administered with OVA, CAF06 effectively activated humoral immunity biased toward Th1 responses [177].

In general, CAF01 is a non-toxic adjuvant that stimulates strong and long-lasting humoral and cell-mediated immune responses. Following intramuscular administration of CAF01, mucosal immune responses are also stimulated [53]. It has reached Phase I clinical trials as a component of vaccines against malaria [184], *Mycobacterium tuberculosis* [185], and HIV [173].

#### 4.2.3. Saponin (QS-21)

**Figure 8:**
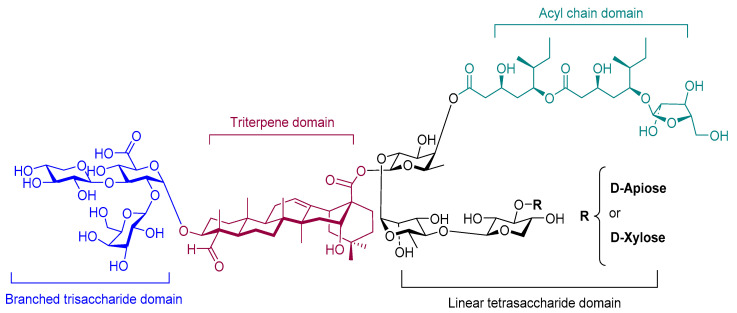
Example of saponin (QS-21) with the four major typical structural domains.

Saponins are complex natural liposaccharides, which have demonstrated an immune stimulatory activity. They can be isolated from the bark, leaf, stem, root, fruit, or flower of more than 100 herbs, including *Saponaria officinalis*, *Quillaja saponaria*, and *Gynostemma pentaphyllum* [186]. The structure of saponins consists of four domains: branched trisaccharide, quillic acid triterpene, binding linear tetrasaccharide, and pseudodimeric acyl chain (Figure 8) [187]. Saponin’s capacity as an immune stimulator has been recognized for 90 years [188]. Its immune-stimulatory properties can be related to the fucosyl residue, which can bind to lectin DC-SIGN on DCs [25]. Consequently, the number, nature, and connectivity type of the glycosyl group of the sugar chains all influence its adjuvanticity [189]. For instance, saponin activity increases when the number of sugars is reduced at the C-3 position or increased at the C-28 position [190].

The most widely utilized plant saponin with adjuvanting activity is QS-21. QS-21, similar to other saponins, can be recognized by lectin receptors on APCs [152]. QS-21 induces CTL activation, Th1 cytokine production, (IL-2 and IFN-γ), antigen-specific antibody responses, Th1 and Th2 immune responses, and antigen cross-presentation [191]. QS-21 is accumulated in lysosomes and causes lysosomal membrane permeabilization, followed by antigen release in the cytosol for further processing and cross-presentation [192]. Hence, the proteasome-independent processing pathway for cross-presentation can be triggered [189,193].

Several studies have been performed to analyze the immune-stimulatory activity of QS-21. One of the oldest malaria vaccine candidates, SPf66, a synthetic 45 amino acid-long peptide derived from four *Plasmodium falciparum* proteins [194] demonstrated poor efficacy when tested with alum [195]. Subsequently, to enhance the safety, tolerability, and immunogenicity of the vaccine, the alum was replaced with QS-21. Although two patients developed severe allergic reactions after the third dose, the SPf66-QS-21 vaccine was found to be safe in 97.8% cases. QS-21 induced anti-SPF66 IgG titers 45- and 200-fold higher than alum-adjuvanted SPf66 after first and second boosts, respectively. QS-21 elicited both antigen-specific CD4^+^ and CD8^+^ T-cell responses, while alum stimulated only CD4^+^ T-cell activation. However, other studies showed that QS-21 in humans caused heterogeneity, scarcity, and dose-limiting toxicity (as flu-like symptoms) after vaccination doses above 50 µg [196]. Therefore, a variety of potentially less toxic simplified QA-21 analogues were prepared [197]. These included a QS-21 derivative that lacked the C4-aldehyde substituent in combination with OVA, which induced the same level of anti-OVA IgG responses as QS-21 in mice but did not trigger weight loss or mortality.

Modification of QS-21 can alter the immune signaling pathway. Myelin oligodendrocyte glycoprotein (MOG35-55) was administered with QS-21 or the deacylated form of QS-21 (QT-0101) into mice [198]. QS-21 + MOG35-55 increased CD4^+^, CD8^+^ T-cells, and IL-17 cell populations, whereas QT-0101 significantly increased IL-4^+^ and CD25/FoxP3^+^ cells and caused T-cell activation and differentiation to Th2 and Treg cells.

Similar to other liposaccharides, QS-21 can be easily incorporated into liposomes. A combination of protein (basic Fibroblast Growth Factor) and QS-21 in liposomal formulation triggered greater IFN-γ and antigen-specific CTL responses than liposome-antigen or saponin-antigen mixtures [199]. Welsby and coworkers demonstrated that the presence of cholesterol in QS-21 liposomal formulation is crucial for its optimal performance, as QS-21 is endocytosed in a cholesterol-dependent manner [192].

Saponins have been used as a major component of a variety of commercial adjuvants. Two commonly used adjuvants, immune-stimulating complexes (ISCOMATRIX) and Quil A, consist of saponins from the South American tree, *Quillaja saponaria* Molina [200]. Other commercial adjuvants bearing saponin include AS01 (liposome, MPLA, and QS-21) [3], AS03 (oil-in-water emulsion, MPLA, and QS-21), and AS15 (liposome, MPLA, QS-21, and CPG) [159]. A variety of vaccines consisting of these adjuvants have been investigated in clinical trials for the prevention/treatment of cancer [201], HIV [202], and hepatitis B [203]. QS-21 is also part of the approved malaria vaccine [204].

##### 4.2.4. α-Galactosylceramide

**Figure 9:**
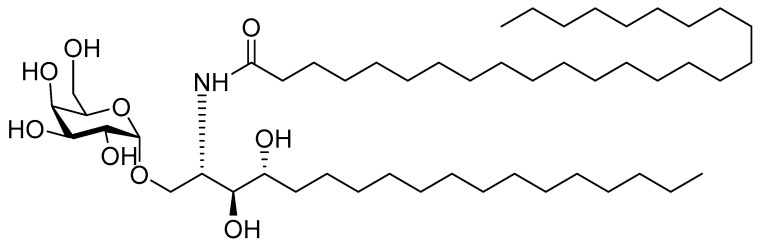
The chemical structure of α-GalCer.

α-galactosylceramide (α-GalCer, Figure 9) is a glycolipidic adjuvant extracted from marine sponges. It is recognized by natural killer T-cells (NKT) as an antigen. α-GalCer can be presented by APCs via the MHC I-like molecule CD1 to NK cells, leading to pro-inflammatory and immune modulatory cytokine responses [205,206]. Activated NKT cells can, in return, activate DCs and enhance responses of antigen-specific CD4^+^ and CD8^+^ T-cells. Thus, it is used as an adjuvant in antiviral and antitumor vaccine design [207]. The conformation and structure of the sugar moiety, as well as the lengths of the fatty acyl chains of glycoceramide, play a significant role in its immunogenicity [205].

The immune-stimulatory capacity of α-GalCer was tested in combination with HIV CTL epitopes derived from gp120 envelope protein [54]. The vaccine candidate was administered in mice through intranasal and oral routes. Mice produced a high level of IFN-γ in the spleen, as well as in mucosal tissues, following both immunizations. Therefore, this adjuvant was considered effective in priming not only systemic but also mucosal immunity. The adjuvant also had the ability to stimulate humoral immune responses. High IgG titers (both IgG1 and IgG2a) were detected following intranasal administration of α-GalCer and OVA, suggesting that α-GalCer induced balanced Th1/Th2 immune responses in mice [208]. OVA-specific mucosal IgA was also produced and the number of IFN-γ-producing CD8^+^ T-cells was significantly higher in all groups immunized with OVA and α-GalCer in comparison with OVA alone. In addition, α-GalCer activated CTLs in both local and systemic lymphoid organs. When challenged with EG7 tumor cells, the formation of tumors was completely blocked in mice immunized with high doses of OVA and α-GalCer. α-GalCer was also tested against viral infection. Mice were intranasally immunized with hemagglutinin from influenza virus alone or with α-GalCer. The vaccine induced strong mucosal immune responses (IgA production), as well as systemic IgG antibody responses, against virus-derived antigens.

A-GalCer has also been tested in combination with other adjuvants. Lipid-polymer nanoparticles (~130 nm, −5 mV) made of PLGA, PLGA-PEG, 1-palmitoyl-2-oleoyl-sn-glycero-3-phosphocholine, and dimyristoylphosphatidylglycerol were loaded with α-GalCer, TLR agonists (MPLA, CpG), and melanoma-associated peptide antigens [209]. The α-GalCer nanoparticles induced a high antigen-specific T-cell response and increased Th1 and Th2 cytokine production. In addition, the combination of α-GalCer and TLR agonist-adjuvanted nanoparticles produced the highest level of infiltration of NK, NKT, and T-cells (CD4^+^ and CD8^+^). The therapeutic antitumor activity of these vaccine candidates was examined in mice inoculated with B16F10 cells at day 0 and immunized with nanoparticles at day 3 and 13. The tumor size in mice immunized with the vaccine candidate bearing α-GalCer, CpG, and MPLA with either mixed or entrapped antigens was 5-fold lower than those of mice immunized with nanoparticles without GalCer. In addition, the nanoparticles bearing α-GalCer/MPLA and entrapped antigens activated NK, NKT, CD4^+^, and CD8^+^ T-cells more efficiently than the mixed formulation. Ultimately, the combination of the NKT cell activator, α-GalCer, and TLR agonists displays synergistic anti-tumor activity.

#### 4.2.5. Muramyl Dipeptide (MDP)

**Figure 10:**
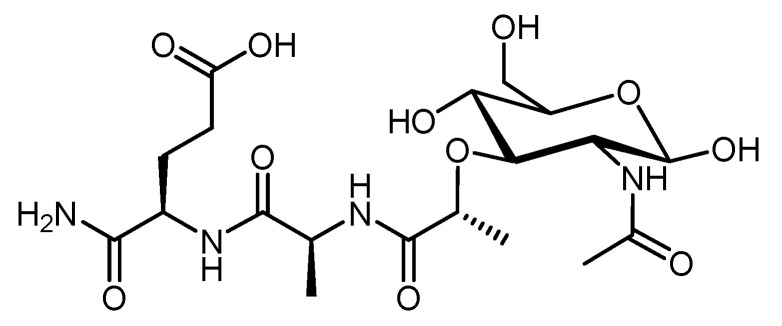
Example of muramyl dipeptide (MDP).

Carbohydrate-peptide conjugates have also been shown to have an adjuvanting capacity. The peptidoglycan *N*-acetyl-muramyl-l-alanyl-d-isoglutamine (muramyl dipeptide, MDP, Gerbu adjuvant) is naturally derived from Gram-positive and Gram-negative bacteria cell walls, but has also been produced synthetically (Figure 10). It is recognized by PRRs, such as NLRs, on APCs [210]. Upon binding to NLRs, MDP causes pro-inflammatory cytokine (e.g., TNF-α, IL-1) and co-stimulates molecule (e.g., IL-6, IL-12) production by APCs and activates both humoral and cellular immunity [211]. While it is not recommended for human vaccines due to its pyrogenic effects, MDP may still be useful for animal vaccines [212]. For instance, MDP with inactivated porcine epidemic diarrhea virus (PEDV) administered subcutaneously to mice induced PEDV-specific IgG antibody and cytokine production and activated CD3^+^ and CD4^+^ cells. The use of MDP in the vaccine also significantly increased PEDV-specific IgA antibody titers following intranasal immunization [213].

Interestingly, the immune-stimulatory capacity of MDP can be further enhanced through a combination with TLR agonists [151]. RSV virosomes, carrying TLR-2 ligand Pam_3_CSK_4_ and MDP modified with C18 fatty acid (L18-MDP), were more efficient than the MDP-adjuvanted vaccine in NF-κB activation, induction of innate and adaptive immune responses (IgA and IgG production), and Th1-skewed responses [214]. Similarly, Pam3CSK4 and MDP chemically conjugated to antigenic peptide with a class I epitope sequence based on OVA stimulated a proinflammatory Th1-type response and CTL activation, as measured by the increase in the level of cytokines (IFN-γ, TNF-α, and IL-2) [215]. This confirmed that MDP mixed with additional adjuvants is able to induce humoral, as well as cellular immunity, in an administration route-dependent manner.

To improve immunogenicity, MDP can be entrapped into nanoparticles. TMC nanoparticles (300–400 nm, 13–21 mV) incorporating OVA and immunopotentiator LPS, Pam_3_CSK_4_, CPG, MDP, or CTB were intradermally or nasally administered to mice [122]. Intradermal administration did not induce detectable IgA production, whereas nasal vaccination with nanoparticles containing LPS, MDP, and CpG resulted in high IgA titers. Nasally administered nanoparticles bearing LPS and MDP induced higher IgG and IgG1 titers than plain nanoparticles, while CPG and LPS nanoparticles were more effective following intradermal administration. Other studies, where vaccines containing chitosan-based particles, MDP, and *Helicobacter pylori* urease (as an antigen) have also shown strong systemic and mucosal immune responses following intranasal administration [210]. While effective, the strength and quality of MDP immune responses are dependent on both the adjuvant and the route of administration.

## 5. Conclusions

Proper adjuvant selection is crucial for stimulating the desired immune response pathways against a targeted pathogen; carbohydrate-based immune stimulants represent a promising emerging group of adjuvants. The number and nature of glycosyl bonds, conformation, the branching degree of polysaccharides, charge, and conjugated moieties all play important roles in carbohydrate adjuvanting potency, but also affect their toxicity. While carbohydrates have a known toxicity (e.g., lipid A), modification of these compounds can often allow for the production of safe, effective derivatives (e.g., MPLA, QS-21). In addition to targeting APC receptors, carbohydrates can also form nanoparticles, adhere to mucosal surfaces, and protect antigens against degradation, allowing them to serve as both adjuvants and delivery systems. Yet, their immune stimulating properties are not fully understood.

Carbohydrates can be especially effective in combination with other adjuvants that target different immune stimulation pathways. Such combinations have already been commercialized (e.g., AS03, AS15), tested in clinical trials (e.g., CAF01), and even utilized in approved human vaccines (e.g., AS01). Interestingly, even carbohydrate adjuvants that are relatively ineffective on their own can be crucial for vaccine efficacy when mixed with other immune stimulators.

In conclusion, the clinical translation of new vaccines against infection and cancer may be facilitated by carbohydrate-based immune stimulators. To develop more effective vaccines, carbohydrates should be considered in combination with other adjuvants, in chemically modified forms, or through functionalization, to stimulate a broader range of immune pathways balanced with safety. The advantageous biocompatibility, biodegradability, mucoadhesivity, common use in pharmaceuticals, and lower cost of production all support further research efforts in the field of carbohydrates for vaccines.
